# Comparing protein structures with RINspector automation in Cytoscape

**DOI:** 10.12688/f1000research.14298.2

**Published:** 2018-06-22

**Authors:** Guillaume Brysbaert, Théo Mauri, Marc F. Lensink

**Affiliations:** 1 CNRS UMR 8576 UGSF, University of Lille, Lille, F-59000, France

**Keywords:** rinspector, cytoscape, protein structure, residue interaction network, centrality analysis, flexibility prediction, automation, structure ensemble

## Abstract

Residue interaction networks (RINs) have been shown to be relevant representations of the tertiary or quaternary structures of proteins, in particular thanks to network centrality analyses. We recently developed the RINspector 1.0.0 Cytoscape app, which couples centrality analyses with backbone flexibility predictions. This combined approach permits the identification of crucial residues for the folding or function of the protein that can constitute good targets for mutagenesis experiments. Here we present an application programming interface (API) for RINspector 1.1.0 that enables interplay between Cytoscape, RINspector and external languages, such as R or Python. This API provides easy access to batch centrality calculations and flexibility predictions, and allows for the easy comparison of results between different structures. These comparisons can lead to the identification of specific and conserved central residues, and show the impact of mutations to these and other residues on the flexibility of the proteins. We give two use cases to demonstrate the interest of these functionalities and provide the corresponding scripts: the first concerns NMR conformers, the second focuses on mutations in a structure.

## Introduction

Knowledge of the structures of proteins is important to understand their function and provide a starting point for further exploration through techniques such as molecular dynamics or docking. These structures can be used directly, in the form of a PDB file, or undergo transformations prior to analysis. Residue interaction networks (RINs) are networks built from a 3D structure, where nodes represent amino acids and edges represent detected interactions between them. These networks can be generated with different tools, like RING2 ^1^, RINerator ^2^ or Chimera ^3^, and then be imported into network visualization and analysis tools. Depending on the purpose, these network tools can be libraries available for languages like R or Python (e.g iGraph or NetworkX) or software with a graphical interface like the well-known Cytoscape ^4^, a reference for biological network studies. The CyREST technology ^5^, which is now included as a core app of Cytoscape 3.3 (and higher versions), enables further analyses, letting the user the possibility to complement analyses done in Cytoscape with scripting developments through external languages like R or Python.

We recently developed the RINspector 1.0.0 ^6^ app for Cytoscape, which combines centrality analyses of residue interaction networks with flexibility predictions of a protein from its associated sequence through a call to the DynaMine flexibility prediction server ^7, 8^. Centrality analyses have been shown to identify residues important for functions, folding or allostery, as well as long range interactions (e.g. Refs 9, 10). Coupled with flexibility predictions, the app enables users to highlight a subset of these central residues, which might affect the dynamics upon mutation ^6^.

The RINspector 1.0.0 app is convenient if one works on only a few networks, but certain centrality calculations may be CPU- and memory-intensive and require long execution times for analyses as soon as the number of networks increases. This may be the case for residue centrality analyses (RCAs) calculated on NMR data, when several conformers need to be considered. Another example is the comparison of RINs generated from a wild-type structure and several mutants. Furthermore, the app provides a score for each residue in each network and the export of a recap chart would be useful to compare between RINs. RINspector now embeds a documented application programming interface (API) in its 1.1.0 version that provides automation of centrality calculations and flexibility predictions, thereby addressing these issues.

Here we present the automation functionalities of RINspector 1.1.0, which allows for combination of the power of Cytoscape with scripting languages such as R or Python, in order to calculate centralities, predict flexibilities, visualize the networks, and get a recap chart with scores. We present two use cases, one based on NMR data and another one based on a mutated domain structure.

## Methods

### Implementation

RINspector 1.1.0 is implemented in Java as an app for Cytoscape 3. It uses the JFreeChart library (Copyright 2000–2009, Object Renery Limited and Contributors) for graph representations. The DynaMine flexibility predictions are retrieved through a JSON API. Betweenness and closeness centrality values were calculated through the functions developed by Assenov *et al.* ^11, 12^ for the Cytoscape NetworkAnalyzer core tools.

Centrality analyses and flexibility predictions can be performed via the Cytoscape graphical interface through the App/RINspector menu (see Ref. 6 for a description). They can also be called through the Cytoscape command dialog or through the CyREST command API presented here.

The RINspector 1.1.0 API relies on the REST (Representational State Transfer) architecture provided by the CyREST tools included in Cytoscape 3.3.0 (or higher), which effectively runs a web service that can only be queried on the local machine that runs Cytoscape. Communication requests follow HTTP protocol and are formatted in JSON.

### Operation

The API consists of two commands, offered with documentation through the CyREST command API in Cytoscape. The commands are:

- ‘centrality’, which calculates centralities for each residue in the residue interaction network currently selected in Cytoscape. This command needs one parameter, which is the type of centrality calculation to perform, selectable between:
  ○ RCA (average shortest path length (ASPL) change under removal of individual nodes)
  ○ Betweenness centrality analysis – BCA
  ○ Closeness centrality analysis – CCA
RCA corresponds to the calculation proposed by 9. The ASPL of the RIN is first calculated, after which the ASPL is calculated for each network upon removal of individual single nodes. A Z-score is then computed for each node based on the change of ASPL compared to the initial one. BCA and CCA are the classical betweenness and closeness centrality calculations, both of which are followed by the calculation of a Z-score (for more details about the process, see the Supplementary materials of Ref. 6).
- ‘dynamine’, which queries the DynaMine server ^7, 8^ with the sequence of the currently selected RIN. This function requires that the table of nodes in Cytoscape contains three columns: ResType, ResIndex and ResChain. The ResType column should contain the 3-letter code for each residue (e.g. ARG). The ResIndex column should contain the serial number (or index) of each residue (e.g. 153). The ResChain column should contain the chain identifier (e.g. A). These three columns are automatically created if the RIN is generated with Chimera through the structureViz2 app. One parameter has to be specified to the ‘dynamine’ command, namely the chain of the protein, formatted as for the ResChain column. The DynaMine server returns a S² flexibility score for each residue.

**Figure 1.**
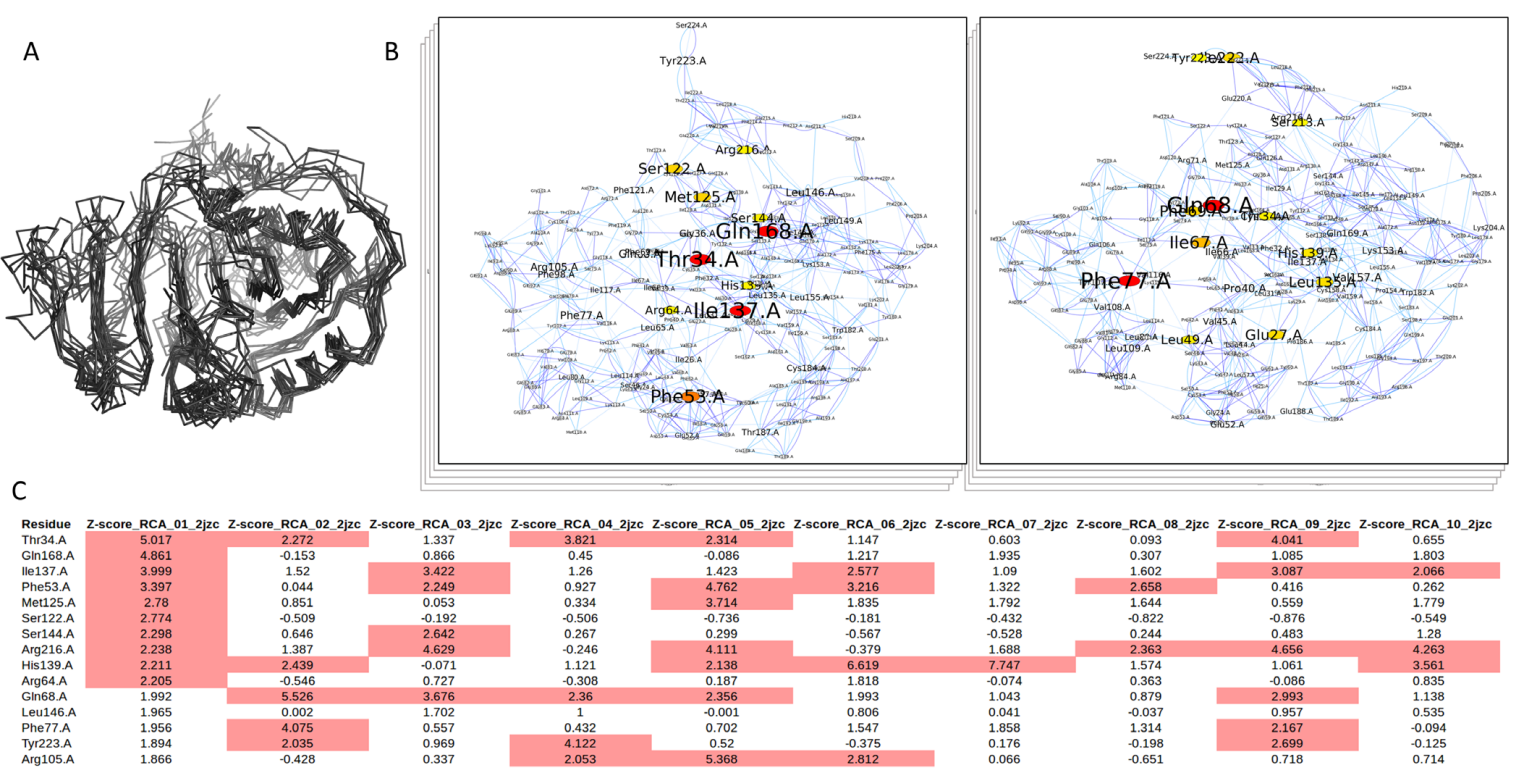
Residue centrality analyses (RCAs) of NMR conformers. ( **A**) Overlapped structure of the 10 conformers used to generate the residue interaction networks (RINs) for ALG13 (PDB ID 2JZC) in ribbon representation. ( **B**) The RINs on which RCAs were performed; central residues are colored from yellow (Z-score = 2) to red (Z-score ≥ 4), labels and node sizes are adapted as functions of the Z-scores: the higher, the bigger; only the first two RINs of the ten calculated are shown. ( **C**) Final result table, which contains the centralities of all the networks, sorted by descending order of Z-scores of the first conformer; Z-scores considered as relevant (Z-score ≥ 2) have a pink background; only the first 15 residues are displayed.

Each run of centrality calculation or flexibility prediction returns a score per residue. These scores appear in a dedicated column in the node table in Cytoscape and are returned as a two-columns table (node ID and score) in JSON through the REST service. The visual style of the network is also adapted (see Figure 1 and Ref. 6). The output table and/or the created columns in the node table can be further treated by a third party program written, for example, in R or Python. Table 1 presents the parameters of the POST request that are used in the provided scripts for a call of centrality or flexibility predictions and associated responses. Considering system requirements, use of a computer with at least 16 GB of memory is advised because the RCAs are memory demanding, and the bigger the structure, the more memory required. The running of use cases should not last more than a few minutes (on a Intel(R) Core(TM) i7-7820HQ CPU @ 2.90GHz, 32Go RAM, 1TB SSD, for RCA: 3m20s for use case 1, 1m47s for use case 2 ; for BCA: 10s for use case 1, 20s for use case 2; for CCA: 7s for use case 1, 17s for use case 2). Automation for RINspector requires Cytoscape 3.6.0 (or higher) and RINspector 1.1.0. The scripts we provide were developed and tested for Python 3.5.2 and R 3.2.3, with Cytoscape 3.6.1.

**Table 1.** Parameters and responses for centrality and dynamine POST requests. For each request to the REST service, the url, body and encoding format are specified. The response in both cases is a table that contains the Z-score (centrality) or the S² score (dynamine) associated to each node ID, in JSON format.

Centrality	dynamine
url: http://localhost:1234/v1/commands/rinspector/centrality body: one of the three methods (RCA/BCA/CCA) encoding: JSON	url: http://localhost:1234/v1/commands/rinspector/dynamine body: chain ID encoding: JSON
response: { "data": { "308053": -0.10838826158689033, "308054": -0.8808526864488292, (…) "308253": 0.9250226566444141 }, "errors": [] }	response: { "data": { "308053": 0.737, "308054": 0.73, (…) "308253": 0.75 }, "errors": [] }

## Use cases

We present here two examples that benefit from the API. The first one considers NMR data with 10 conformers of a yeast N-acetylglucosamine transferase, all grouped in the PDB ID 2JZC. The second one is the tetratricopeptide repeat (TPR) domain of a human O-GlcNAc transferase (OGT) (PDB ID 4GYW). Use cases files including scripts, inputs and results are available in the ‘ use_cases+scripts’ folder of the git repository. README.txt files in each use case folder detail the steps to follow to run them.

### Use case 1: 2JZC

NMR data are well suited for automation treatment because the associated PDB files usually contain multiple conformers for which RINs can be generated. We built the RINs in Cytoscape from Chimera through the structureViz2 app. Once these are created, the RINspector API can be queried to calculate centralities in batch. In this example, we calculate residue centrality scores for each residue and compare them between the conformations. We wrote a script in R and an equivalent one in Python that, starting from a Cytoscape session containing one RIN for each conformer, perform RCA on each RIN. The scripts then gather the Z-scores for each residue in each RIN in a single recap table, allowing for easy comparison. The app also creates a style for each RIN that permits a visual comparison ( Figure 1).

### Use case 2: 4GYW

Here we compare the centralities of residues when RINs are generated from structures which contain point mutations. We also compare the impact of mutations on flexibility.

**Figure 2.**
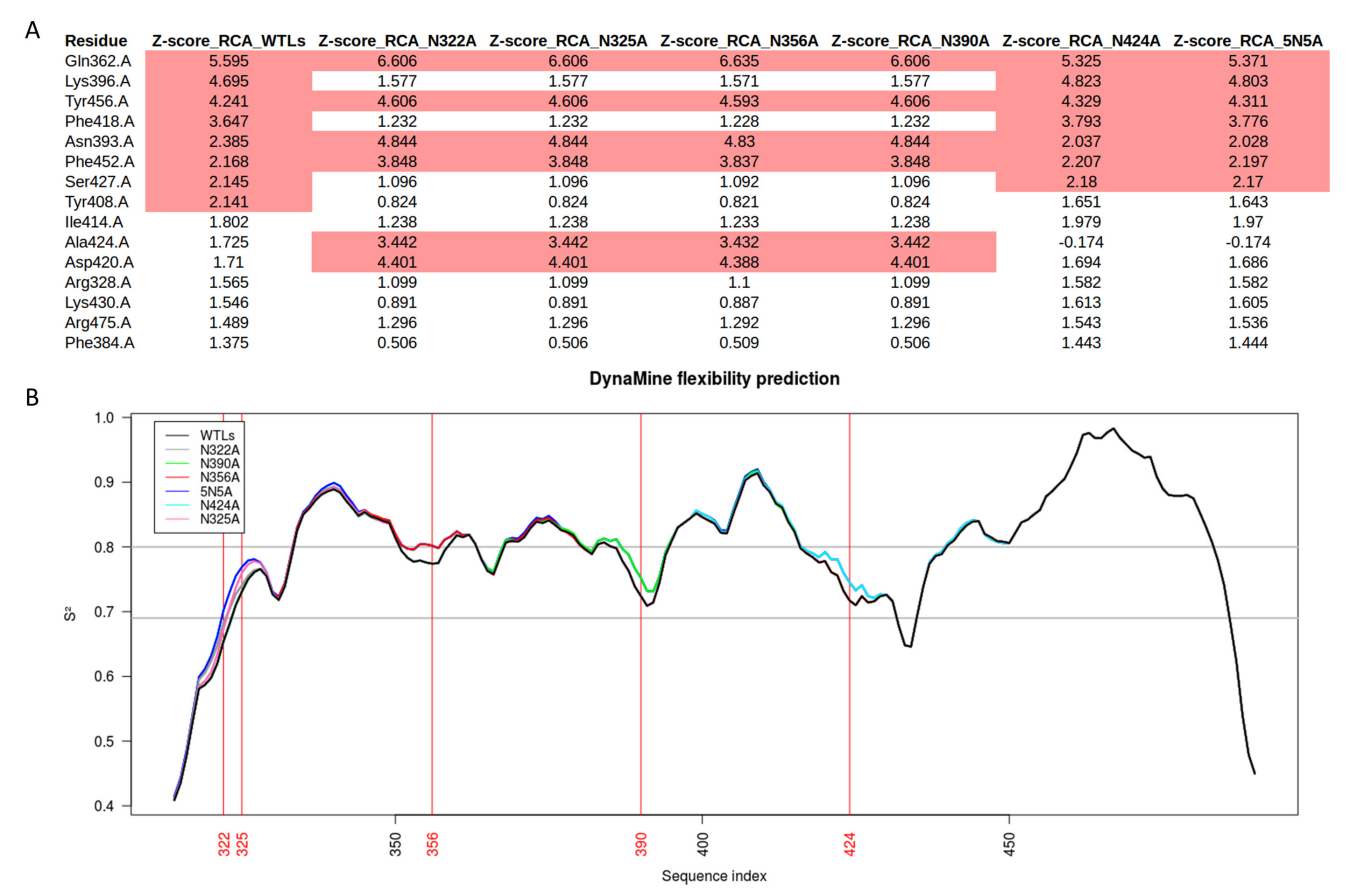
Residue Centrality Analyses (RCAs) of the TPR domain of the human O-GlcNAc transferase (PDB ID 4GYW). ( **A**) Final result table showing the wild type TPR domain of the human OGT and the 6 mutants N322A, N325A, N342A, N356A, N390A and 5N5A (containing all five mutations), sorted by descending order of Z-scores of the wild type; Z-scores considered as relevant (Z-score ≥ 2) have a pink background; only the first 15 residues are shown. ( **B**) DynaMine flexibility graphs of the wild type (WTLs) and each of the mutants; X=sequence index, Y=S², the higher S² value, the more rigid the backbone is predicted to be; grey horizontal straight lines delimit a context dependent zone; mutated residues are highlighted with red vertical straight lines.

We created six mutants in the tetratrico peptide repeat (TPR) domain of the O-GlcNAc transferase (OGT) by editing the PDB file of the wild type. Five mutants were asparagines to alanines (N322A, N325A, N356A, N390A, N424A), the sixth contained all five point mutations. The OGT is an enzyme that catalyzes the transfer of a single N-acetylglucosamine from UDP-GlcNAc to a serine or threonine amino acid (called O-GlcNAcylation). These five asparagines have been shown to decrease the efficiency of the OGT enzymatic activity on a category of peptides when simultaneously mutated into alanines ^13^. We generated the RINs (without ligands) for the wild-type structure and each of the six variants. We wrote a script that, starting from a Cytoscape session containing these RINs, calculates residue centralities and predicts flexibilities for each structure. As in the use case 1, the results of centralities are gathered in a common table. In addition, the S² flexibility scores are also gathered in a recap table and in a single plot to permit easy comparison ( Figure 2). These tables and plot allow for comparison between the centralities, to see which of the mutations have an impact on which centralities compared to the wild type, and to see the impact of each mutation on the backbone flexibility of the TPR domain.

## Discussion

Comparisons between structures, and more precisely between residues in structures, are useful for identification of one or a subset of amino acids that are crucial for the folding or functions of proteins. The automation functionalities of RINspector 1.1.0 provided through CyREST in Cytoscape make these comparisons easier. Indeed, they permit to perform centrality analyses and flexibility predictions of residue interaction networks generated from structures of proteins, in batch mode from an external script. The results can then be exploited using the libraries available for the language of the script (usually R or Python).

In our cases, we treated at most ten networks at a time, which is reasonable. This number can of course be higher, but depending on the size of the structure/network, it may be difficult to deal with a substantially higher number of networks. Indeed, while BCAs and CCAs are usually performed within a few seconds, RCAs are memory demanding and running many of them may quickly fill the RAM of the system. In such cases we recommend to perform all the calculations through a workflow outside Cytoscape. The CyREST core or command API can nevertheless still be called in the workflow, e.g. for the generation of networks visualizations. RCAs and BCAs usually give many central residues which show various degrees of overlap. Depending on the objective of the analyses, it may be more relevant to consider the results of one centrality, the intersection, or the union of these selections. However, CCAs generally result in few residues with Z-scores ≥ 2. If the user is interested in this specific measurement, we advise to visualize directly the closeness centrality values and not the Z-scores (with NetworkAnalyzer tools in Cytoscape or RINalyzer app ^2^ for instance).

The RINspector 1.1.0 API can be used in conjunction with other apps, especially with the structureViz2 app, which connects Chimera to Cytoscape, because it permits to open a structure in Chimera and work with it. This connection allows for the easy generation of RINs and the possibility of synchronization of colors between network and structure, which is a welcome feature for the generation of images. In this respect we would also like to point out the RINalyzer app, which enables the user to layout the RIN in function of coordinates of residues displayed in Chimera.

In the use case 2, DynaMine results were exploited to build a chart for comparison of flexibilities between structures. For such analyses the proposed script is particularly interesting, as computation resources are not a limiting factor. In most cases, however, only a few mutations are really of interest and the result panel designed for DynaMine in RINspector may be more convenient to visualize the effect of these mutations directly and select one or several for protein design. The user should also be aware that the sequence sent to the DynaMine server is built from the RIN/structure, which means that missing residues will simply be skipped in the DynaMine flexibility graph.

Other use case examples might be the effect of one or several ligands in a structure by generating RINs with or without these ligands, the comparison of centralities of different structures of the same complexes or the comparison of the flexibility of structures of several orthologs. Our scripts constitute starting points to perform such analyses with recap tables and charts as output.

### Future developments

We plan to extend the interaction between other languages and Cytoscape complementing batch centrality analyses and flexibility predictions with automatic generation of RINs and integration of conservation data.

## Summary

The RINspector 1.1.0 API permits scripting of centrality analyses and flexibility predictions. The automation provided bridges between Cytoscape and other languages, such as R or Python, to prepare data, run batch analyses and treat output data. It enlarges the possibilities of treatments that were initially given by the app in particular to compare centralities and flexibilities between multiple structures. We provide R and Python scripts that illustrate two use cases and that are to be seen as starting scripts for more elaborate analyses.

## Data and software availability

1. RINspector is available from the Cytoscape App Store: http://apps.cytoscape.org/apps/rinspector

2. Latest source code and example files are available at: https://sourcesup.renater.fr/scm/?group_id=3888

3. Archived source code as at time of publication: https://doi.org/10.5281/zenodo.1292457 ^14^.

4. Software license: CeCILL; version 2.1: http://www.cecill.info
