# Supplementary material for: Comparing protein structures with RINspector automation in Cytoscape
Source: F1000Res. 2018 Jun 22;7:563. Originally published 2018 May 9. [Version 2] doi: 10.12688/f1000research.14298.2 (PMC5998007; doi:10.12688/f1000research.14298.2)
Supplement: Peer review history

# Peer review: Referee response for version 1

**DOI:** 10.5256/f1000research.15559.r33873

**Published:** 2018-06-18

**Authors:** Rita Casadio

**Affiliations:** 1 Biocomputing Group, Department of Pharmacy and Biotechnology, University of Bologna, Bologna, Italy

Comparing protein structure with RINspector is an intersting application that allows an easy determination of crucial residues in protein structures. It can be useful  for pinpointing specific positions in the protein backbone for further experimental investigations.

 The paper clearly explains the procedure, and clarifies with test cases the relevance of the application. Software is detailed and reproducible. Figures are clear and appropriate.

I have read this submission. I believe that I have an appropriate level of expertise to confirm that it is of an acceptable scientific standard.

# Response

**Published:** 2018-06-20

**Authors:** Guillaume Brysbaert

**Affiliations:** CNRS, France

Dear Dr Casadio,

 Thank you for the review and your interest in our work. We are happy to see that you accept the manuscript.

 Best regards

# Peer review: Referee response for version 1

**DOI:** 10.5256/f1000research.15559.r33872

**Published:** 2018-06-12

**Authors:** Turkan Haliloglu, Ezgi Karaca

**Affiliations:** 1 Polymer Research Center & Department of Chemical Engineering, Boğaziçi University, Istanbul, Turkey; 2 International Biomedicine and Genome Institute, Dokuz Eylul University, Balcova, Izmir, Turkey

The RINspector app allows network and flexibility analysis of protein structures, which plausibly has the capacity to disclose functionally critical residues. The paper by Brysbaert et al. explains the updated (automated) version of the RINspector app. The new version is stated to work more efficiently than previous one, especially while treating multi-conformers. The paper could further be improved, if the following points are addressed.

 - The differences between the previous and current version of the program are not clearly stated. It is confusing what is new and what is updated, especially for someone who is not familiar with the RINspector app. Maybe the authors could mention the version numbers explicitly when they make a comparison.

 - There is a tutorial provided for the initial version of RINspector. A similar tutorial should also be prepared for the use cases presented in this paper. In this way, the users can exactly reproduce what is stated in the manuscript.

 - Relevant links to the software, the use case folders and scripts should be cited within the text. In the current version, it is hard to link the source code files with the options/conditions stated in the paper.

 Overall, RINspector app stands a useful resource that would corroborate structural and functional studies in the community.

We have read this submission. We believe that we have an appropriate level of expertise to confirm that it is of an acceptable scientific standard.

# Response

**Published:** 2018-06-20

**Authors:** Guillaume Brysbaert

**Affiliations:** CNRS, France

Dear Dr Haliloglu and Dr Karaca,

 Thank you for your interest in our article and your constructive comments. We address them point by point.

 - The differences between the previous and current version of the program are not clearly stated. It is confusing what is new and what is updated, especially for someone who is not familiar with the RINspector app. Maybe the authors could mention the version numbers explicitly when they make a comparison.

 → The versions of RINspector were specified in the text to avoid confusion.

 - There is a tutorial provided for the initial version of RINspector. A similar tutorial should also be prepared for the use cases presented in this paper. In this way, the users can exactly reproduce what is stated in the manuscript.

 → We included a ‘README.txt’ file in each use case folder to explain how to run the example scripts. The point of the paper being the automation of RINspector, we don’t explain how to generate the RINs but we provide a python script called ‘creation_RIN_NMR.py’ in the use case 1 that permits to generate the scripts with structureViz2/Chimera.

 - Relevant links to the software, the use case folders and scripts should be cited within the text. In the current version, it is hard to link the source code files with the options/conditions stated in the paper.

 → In the ‘Use cases’ part, we added the information that the use cases files (inputs/scripts/outputs) are available in the git repository and added a hyperlink to this folder in the git.

 Best regards

# Peer review: Referee response for version 1

**DOI:** 10.5256/f1000research.15559.r33871

**Published:** 2018-06-12

**Authors:** Sjoerd J. de Vries

**Affiliations:** 1 MTi, UMR‐S 973, Physics Department T38, Technical University of Munich, Garching, Germany

Brysbaert et al. describe an API for their RINspector tool for the analysis of residue interaction networks (RINs). This API allows RIN analysis in an automated fashion (batch mode), controlled from any programming language that can perform HTTP requests. The authors provide two use cases as an example.

 The article is sound and well-written. I have only minor comments pertaining the didactic value of the paper: I feel the authors could lower the barrier for users of the software, by explaining better what it is good for and how to get it running.

- The running time of the use cases should be reported, to demonstrate the amount of human time saved.
- The use case example scripts are available from the Git repository, but this is not mentioned, and the repository itself is not easy to browse. Direct links would be better. 
- The software relies heavily on Cytoscape, its app store, and it's cyREST technology, and the authors assume that the reader has good knowledge of these. However, Cytoscape is not much used in the protein structure community. I recommend that the authors include a link to a short install manual for Cytoscape, cyREST and RINspector, plus any other necessary steps that must be performed before the example scripts can be run.
-  It deserves some explanation that the RINspector API is not meant to (and in fact, unable to) provide any kind of web service. The reason why might be well-known within the Cytoscape community, but again, Cytoscape is not very well known among structural biologists, and I have never used it myself. From what I understand, the cyREST technology is very poorly named, since it is not a proper (i.e. stateless) REST protocol at all. Instead, it seems to be a technology to send state-manipulating commands (load data; analyze current data; etc.) over HTTP to a running instance of Cytoscape. The RINspector API follows this state-manipulating paradigm. In addition, it seems that Cytoscape cannot run behind a web server because it always runs a GUI. 

I have read this submission. I believe that I have an appropriate level of expertise to confirm that it is of an acceptable scientific standard.

# Response

**Published:** 2018-06-20

**Authors:** Guillaume Brysbaert

**Affiliations:** CNRS, France

Dear Dr de Vries

 Thank you for your interest of our manuscript and your helpful remarks. We took them in consideration and updated the manuscript. Here are our replies to your comments:

- The running time of the use cases should be reported, to demonstrate the amount of human time saved.

→ Running times were added in the text in the ‘Operation’ part.

- The use case example scripts are available from the Git repository, but this is not mentioned, and the repository itself is not easy to browse. Direct links would be better.

→ We added the information that the example files are available in the git repository. We specified the relative folder on the git repository and set a link to the folder in tree visualisation.

- The software relies heavily on Cytoscape, its app store, and it's cyREST technology, and the authors assume that the reader has good knowledge of these. However, Cytoscape is not much used in the protein structure community. I recommend that the authors include a link to a short install manual for Cytoscape, cyREST and RINspector, plus any other necessary steps that must be performed before the example scripts can be run.

→ In each use case folder, we included a README.txt file that details the required steps to run the scripts. We added this information in the ‘Use cases’ part of the article. CyREST is included in the last versions of Cytoscape, that’s why the README.txt files don’t contain any mention about CyREST installation.

- It deserves some explanation that the RINspector API is not meant to (and in fact, unable to) provide any kind of web service. The reason why might be well-known within the Cytoscape community, but again, Cytoscape is not very well known among structural biologists, and I have never used it myself. From what I understand, the cyREST technology is very poorly named, since it is not a proper (i.e. stateless) REST protocol at all. Instead, it seems to be a technology to send state-manipulating commands (load data; analyze current data; etc.) over HTTP to a running instance of Cytoscape. The RINspector API follows this state-manipulating paradigm. In addition, it seems that Cytoscape cannot run behind a web server because it always runs a GUI.

→ RINspector is an app for the standalone Cytoscape software that uses the REST service provided by the CyREST tool inside Cytoscape. As such, contrarily to the usual REST services designed for a web usage, Cytoscape is a standalone tool with a GUI that has to be run on a single computer to start the REST service that is only accessible on this computer. Requests are done thanks to the HTTP protocol and are formatted in JSON. We added explanations in the ‘Implementation’ part. The REST architecture that is used is stateless in the sense that each request is independent from another and no state is conserved by the local server.

 Best regards
